# Supplementary material for: Validity and reliability of the Sustainable HEalthy Diet (SHED) index by comparison with EAT-Lancet diet, Mediterranean diet in Turkish adults
Source: PeerJ. 2024 Sep 30;12:e18120. doi: 10.7717/peerj.18120 (PMC11448654; doi:10.7717/peerj.18120)

|  | **SHED questions (English)** | **Supplementary Materials** |
| --- | --- | --- |
| scale | Kindly specify to what extent the following sentences are accurate for you personally: | Domain |
| Scale (1-4):  Almost Never True  Rarely True  Often True  Almost Always True | - As a main course, I prefer and eat meat products (poultry, beef, fish) more times per week compared to plant-based food (grains, legumes, fruits and vegetables) | Health:  Healthy Eating (HE)  10 items |
|  | - In a course of a week, I eat more plant-based food (grains, legumes, fruits and vegetables) instead of animal source foods (meat, dairy products and eggs) |  |
|  | - I eat a variety of fruits and vegetables, at least 400 grams or 5 portions daily |  |
|  | - I try to avoid meat and fatty meat products and prefer instead beans, legumes, lentils, fish, poultry or low-fat meat |  |
|  | - I prefer buying and consuming low salt products |  |
|  | - I try to avoid buying and consuming ultra-processed food products |  |
|  | - I prefer drinking water (or carbonated water) as a main beverage |  |
|  | - I choose low-sugar foods |  |
|  | - I limit the frequency of consumption of sweetened beverages and sweets |  |
|  | - I control the amount of salt I consume and limit adding salt to my meals |  |
| Scale (1-4):  Almost Never True  Rarely True  Often True  Almost Always True | - I separate waste and recycle food scraps at home with a composter (tool for producing organic fertilizer from food scraps)* | Environment and socio-economic:  Sustainable Eating  (SE)  7 items |
|  | - I prefer buying and eating food made in Israel as much as possible |  |
|  | - I limit my meat consumption |  |
|  | - I try to eat crops that are reduced or free of pesticides and herbicides |  |
|  | - I try to consume organic food products on a regular basis** |  |
|  | - I am aware and act to reduce food waste in my close environment |  |
|  | - I eat plant-based foods as an alternative to meat on a regular basis |  |
| Multiple choice | *(if yes) Kindly specify type of composter : |  |
|  | - Neighborhood |  |
|  | - Private (backyard) |  |
|  | - Home (indoors) |  |
|  | ** (if yes) Expansion to Organic |  |
| Multiple choice | Kindly specify the type of organic food you consume (more than one answer may be selected) | Socio cultural |
|  | - Vegetables |  |
|  | - Fruits |  |
|  | - Grains and legumes |  |
|  | - Dairy products |  |
|  | - Meat products |  |
|  | - None |  |
| Multiple choice |  | Socio cultural |
|  | - Directly from the farmer |  |
|  | - Small shop in town |  |
|  | - Social business |  |
|  | - Food chain |  |
|  | - Self-grow |  |
|  | - Other |  |
| Scale for each:  Never  Seldom  Some of the time  Most of the time | Where do you buy fruits and vegetables? | Socio-cultural and socio-economic*  (BFV score) |
|  | - Self-grow |  |
|  | - Direct delivery / Box from the farmer |  |
|  | - Buy directly at a farm |  |
|  | - At the market |  |
|  | - At a grocery store, or at a small, non-chain grocery store |  |
|  | - At a country store/green grocery (fruits & vegetables store) |  |
|  | - Supermarket – Home delivery |  |
|  | - Supermarket – Shop in person |  |
|  |  |  |
|  | At what frequency do you: |  |
| Scale for each:  Never  Rarely (up to one time monthly)  Occasionally (once-twice monthly)  Sometimes (about once a week)  Often (two-three times weekly)  Daily or almost daily | - Eat pre-prepared meals - frozen | Socio-cultural and health*  (ready meals score) |
|  | - Eat pre-prepared meals – chilled (packed) |  |
|  | - Eat homemade or home cooked food (not necessarily at your home) |  |
|  | - Eat in restaurants or eateries or cafeteria at work |  |
|  | - Cook food by myself (or take part in preparing it) |  |
|  | - Consume food cooked 1-3 days prior to eating |  |
|  |  |  |
|  | Kindly specify the type of water you drink and frequency: |  |
| Scale for each:  Never  Seldom  Some of the time  Most of the time | - Unflavored Tap water/Homemade carbonated water/SodaStream (Israeli brand of soda water maker) | Environmental  Drinking Habits  (water score) |
|  | - Home water filters (Tami4, Brita, etc.) |  |
|  | - Large bottled water cooler (mineral water) |  |
|  |  |  |
|  | At what frequency do you drink: |  |
| Scale for each:  Never  Rarely (up to once monthly)  Occasionally (once-twice monthly)  Sometimes (about once weekly)  Often (two-three times weekly)  Daily or almost daily | - Soft drinks (for example Coca-Cola, Sprite, Nestea, etc.) | Environmental and health  Drinking Habits  (Soda score) |
|  | - Diet beverages (Diet Coke, Diet Sprite, Coke Zero, Pepsi Max, etc.) |  |
|  |  |  |
|  | **Plant-base self-assessment:** |  |
|  | *Plant-based food includes: Grains, legumes, fruits and vegetables.* |  |
|  | *Animal source food includes: Meat, poultry, fish, eggs, dairy products* |  |
| Percent (%)  0-------------------100 | Based on this definition, what percentage of your diet is based on plant-based foods? Slide to answer |  |
|  |  |  |
|  | **Waste streams**** |  |
| Yes  No | Is (or was) separation of wet (organic) waste customary where you live? | Environmental |
|  |  |  |
| Percent of organic waste separation:  0-------------------100 | What percentage of organic waste did you separate and discard in a designated bin? Slide to answer |  |
|  |  |  |
| Yes  No | Is there an orange trash bin (In Israel – bin for recycling of empty packages except glass or cardboard) where you live? |  |
|  |  |  |
| Percent of package recycling:  0-------------------100 | What percent of packages did you separate and discard in the designated trash bin? Slide to answer |  |
|  |  |  |
| Yes  No | Is there a plastic bottles recycling bin where you live? |  |
|  |  |  |
| Percent of bottles recycling:  0-------------------100 | What percent of plastic bottles did you separate and discard in the recycling bin? Slide to answer |  |
|  |  |  |
| Yes  No | Is there a purple trash bin (In Israel – bin for glass recycling) where you live? |  |
|  |  |  |
| Percent of glass recycling:  0-------------------100 | What percent of glass waste did you discard in the purple bin? Slide to answer |  |

Table 2. Mediterranean Diet Adherence Screener (MEDAS)
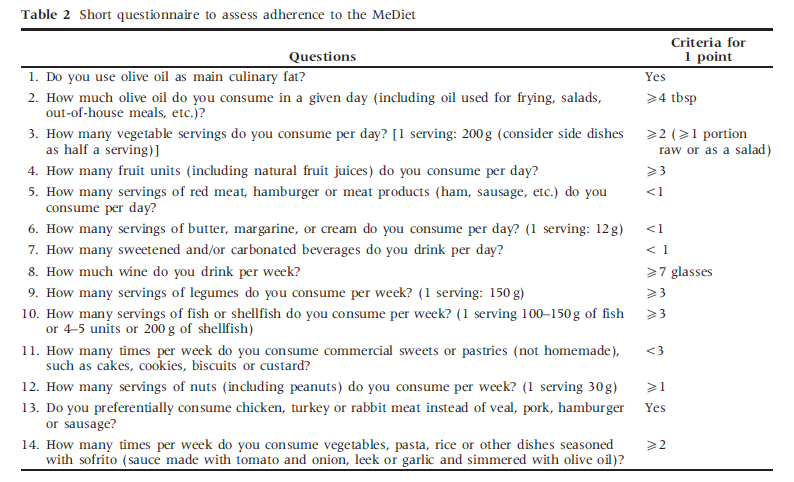

Supplement: Supplemental Information 3 [file peerj-12-18120-s003.docx]
