# Supplementary material for: Validity and reliability of the Sustainable HEalthy Diet (SHED) index by comparison with EAT-Lancet diet, Mediterranean diet in Turkish adults
Source: PeerJ. 2024 Sep 30;12:e18120. doi: 10.7717/peerj.18120 (PMC11448654; doi:10.7717/peerj.18120)
Supplement: Supplemental Information 4 [file peerj-12-18120-s004.docx]

| **SAĞLIK**  **Health/ Healthy Eating**  **Beslenme Değişkenleri ( 10 madde)** | Benim için neredeyse hiç geçerli değil | Benim için nadiren  geçerli | Benim için genellikle/sıklıkla  geçerli | Benim için neredeyse her zaman geçerli |
| --- | --- | --- | --- | --- |
| Hafta boyunca, ana yemeklerimde et ürünlerini (kırmızı et, tavuk, hindi ve balık) bitkisel besinlere (tahıllar, baklagiller, meyve ve sebzeler) göre/kıyasla daha fazla tercih ederim ve yerim. | 3 | 2 | 1 | 0 |
| Hafta boyunca, hayvansal kaynaklı besinler (et, süt ürünleri ve yumurta) yerine daha çok bitkisel kaynaklı besinler (tahıllar, baklagiller, meyveler ve sebzeler) yerim. | 0 | 1 | 2 | 3 |
| Bir gün boyunca toplamda en az 400 gram veya 5 porsiyon çeşitli meyve ve sebze yerim. | 0 | 1 | 2 | 3 |
| Et ve yağlı et ürünlerinden (koyun, dana eti, sakatat ve şarküteri ürünleri (sucuk, salam gibi) uzak durmaya çalışır ve bunun yerine fasulye, baklagil, mercimek, balık, kümes hayvanları veya az yağlı etleri (hindi, tavuk) tercih ederim. | 0 | 1 | 2 | 3 |
| Tuz içeriği düşük ürünleri tercih ederim ve satın alırım.  Jambon, sucuk, pastırma gibi şarküteri ürünleri, hazır et suyu veya tabletleri, soya sosu gibi sosları, cips, kraker, turşu veya turşu sularını ve diğer tuz içeriği yüksek ürünleri tercih etmem ve satın almam. | 0 | 1 | 2 | 3 |
| Ultra işlenmiş yiyecekleri (hazır kek, çikolata, cips, gofret, şekerlemeler, bisküvi, sosis, salam, sucuk, fast-food gıdalar) satın almaktan ve tüketmekten kaçınırım. | 0 | 1 | 2 | 3 |
| Genel olarak tüm içecekler düşünüldüğünde, en fazla içme suyu veya maden suyu içerim. | 0 | 1 | 2 | 3 |
| Şeker içeriği düşük yiyecekleri tercih ederim.(Meyveler dahil değildir) | 0 | 1 | 2 | 3 |
| Kola, gazoz, hazır meyve suyu, soğuk çay gibi şekerli içecekleri ve tatlıları nadiren, sınırlı tüketirim. | 0 | 1 | 2 | 3 |
| Tuz tüketimimi kontrol ederim ve yemeklerime tuz eklemeyi sınırlandırırım (Örn. Yemeklerime en fazla 5 gram tuz eklerim gibi bir sınırlama) | 0 | 1 | 2 | 3 |

| **ÇEVRE ve SOSYO-DEMOGRAFİK**  **Sürdürülebilir Beslenme (7madde)**  **Sustainable Eating (SE):** | Benim için neredeyse hiç geçerli değil | Benim için nadiren geçerli | Benim için genellikle/sıklıkla  geçerli | Benim için neredeyse her zaman geçerli |
| --- | --- | --- | --- | --- |
| Evde bir kompost makinesi (yemek artıklarından organik gübre üretme aracı) ile atıklarımı ayırırım ve yemek artıklarını geri dönüştürürüm. | 0 | 1 | 2 | 3 |
| Mümkün olduğunca Türkiye’de yetişen besinleri ve üretilen gıdaları satın almayı ve tüketmeyi tercih ederim. | 0 | 1 | 2 | 3 |
| Et tüketimimi sınırlandırırım/sınır koyarım (Örneğin haftada toplam 150 gram) | 0 | 1 | 2 | 3 |
| Tarım ve böcek ilacı içermeyen veya az içeren bitkisel besinleri tüketmeyi tercih ederim. | 0 | 1 | 2 | 3 |
| Organik besinleri düzenli tüketmeye çalışırım. | 0 | 1 | 2 | 3 |
| Gıda israfının farkındayım ve yakın çevremde azaltmak için çaba gösteriyorum | 0 | 1 | 2 | 3 |
| Düzenli olarak et tüketmek yerine bitkisel besinleri ( mercimek nohut gibi baklagiller, meyve ve sebzeler) tüketmeye çalışırım | 0 | 1 | 2 | 3 |

| **SOSYO KÜLTÜREL** | Hayır | Evet |
| --- | --- | --- |
| Eğer kompost makinanız varsa lütfen yanıtlayınız.  *(Birden fazla işaretleyebilirsiniz)* |  |  |
| Kompost makinesi komşularımda var |  |  |
| Evin dışında, bahçemde var |  |  |
| Evin içinde var |  |  |
| Organik besinlerden aşağıdakilerden hangisini tüketiyorsunuz?  *Birden fazla işaretleyebilirsiniz* |  |  |
| Sebzeler |  |  |
| Meyveler |  |  |
| Tahıllar ve baklagiller (buğday, pirinç, mercimek, nohut gibi) |  |  |
| Süt ve ürünleri |  |  |
| Et ve ürünleri |  |  |
| Hiçbiri |  |  |
| Organik besinleri aşağıdakilerden hangisinden satın alırsınız?  *Birden fazla işaretleyebilirsiniz* |  |  |
| Doğrudan üreticiden |  |  |
| Küçük-yerel gıda market/pazarlardan |  |  |
| Zincir marketlerden |  |  |
| Food chain/Gıda/Besin zinciri |  |  |
| Kendim yetiştiririm |  |  |
| Diğer |  |  |

| **SOSYO KÜLTÜREL ve SOSYO EKONOMİK**  **(BFV Skor)** | Hiçbir zaman | Nadiren | Bazen | Genellikle |
| --- | --- | --- | --- | --- |
| Sebze ve meyveleri nereden satın alırsınız? |  | | | |
| Kendim yetiştiririm | 0 | 1 | 2 | 3 |
| Doğrudan üreticiden (çiftlik, tarla, kendi köyünden getirme) sipariş verir kargoyla teslim alırım | 0 | 1 | 2 | 3 |
| Doğrudan çiftlikten kendim gider satın alırım | 0 | 1 | 2 | 3 |
| Marketten satın alırım | 3 | 2 | 1 | 0 |
| Bakkaldan, zincir olmayan bir marketten satın alırım | 3 | 2 | 1 | 0 |
| Manavdan satın alırım | 3 | 2 | 1 | 0 |
| Eve teslimat yapan marketler ve diğer uygulamalardan (Getir, iste gelsin, sanal marketler gibi) sipariş veririm | 3 | 2 | 1 | 0 |
| Süpermarketten kendim alışveriş yaparım | 3 | 2 | 1 | 0 |

| **SOSYO KÜLTÜREL ve SAĞLIK**  **Ready meals score** | **Hiç** | **Nadir (ayda1)** | **Ara sıra (Ayda 1-2 kez)** | **Bazen**  **(Haftada 1 kez)** | **Genellikle**  **(Haftada 2-3)** | **Her gün veya hemen hemen her gün** |
| --- | --- | --- | --- | --- | --- | --- |
| Dondurulmuş hazır/ambalajlı yemekleri ne sıklıkla yersiniz? | **5** | **4** | **3** | **2** | **1** | **0** |
| Ambalajlı hazır yemekleri ne sıklıkla yersiniz? | 5 | 4 | 3 | 2 | 1 | 0 |
| Ev yapımı yemekleri ne sıklıkla yersiniz (evde sizin yapmanız gerekmez/esnaf lokantaları da dahil) ? | 0 | 1 | 2 | 3 | 4 | 5 |
| İş yerinde restoran veya yemekhanede ne sıklıkla yersiniz? | 0 | 1 | 2 | 3 | 4 | 5 |
| Kendi yemeklerimi pişiririm (veya pişirme işlerine dahil olurum) | 0 | 1 | 2 | 3 | 4 | 5 |
| 1-3 gün önceden pişmiş yemekleri tüketirim | 0 | 1 | 2 | 3 | 4 | 5 |

| **Çevre ve SAĞLIK**  **Drinking Habits**  **(water score)** | **Hiç veya nadir (ayda1)** | **Ara sıra (Ayda 1-2 kez)** | **Bazen**  **(Haftada 1 kez)** | **Genellikle**  **(Haftada 2-3)** | **Her gün veya hemen hemen her gün** |
| --- | --- | --- | --- | --- | --- |
| Musluk suyu | **0** | **1** | **2** | **3** | **4** |
| Ev tipi su arıtma cihazı suyu | 0 | 1 | 2 | 3 | 4 |
| Damacana veya şişe suyu (satın alınan içme suyu) | 4 | 3 | 2 | 1 | 0 |
| **Drinking Habits**  **(Soda score)** | | | | | |
| Alkolsüz içecekler meşrubatlar, kola, gazoz, maden suyu, soğuk çay | 4 | 3 | 2 | 1 | 0 |
| Diyet alkolsüz içecekler (kola, gazoz) | 4 | 3 | 2 | 1 | 0 |

| **Bitkisel Beslenme Kişisel Değerlendirme** |  |
| --- | --- |
| Tahıllar, baklagiller, meyveler ve sebzeler bitkisel kaynaklı besinlerdir  Kırmızı etler, beyaz etler, yumurta, balık, süt ve süt ürünleri hayvansal kaynaklı besinlerdir  ***Bu tanımlara göre sizce günlük beslenmenizin yüzde kaçı bitkisel besinlerden oluşuyor?*** | 0-100 |

| **ÇEVRESEL**  **Atık ayrıştırma alışkanlıkları** | Hayır | Evet |
| --- | --- | --- |
| Yaşadığınız bölgede sebze meyve, çay, kahve posası gibi mutfak atıklarının (organik atık) ayrılması uygulanır mı? (uygulanır mıydı)? |  |  |
| Organik atıkların yüzde kaçını ayırıp belirlenmiş bir geri dönüşüm kutusuna attınız? | 0-100 | |
| Yaşadığınız bölgede cam ve karton dışındaki ambalaj atıklar için geri dönüşüm kutusu var mı? | 0-100 | |
| Cam ve karton dışındaki ambalaj atıklarınızın yüzde kaçını belirlenmiş bir geri dönüşüm kutusuna attınız? | 0-100 | |
| Yaşadığınız yerde plastik şişeler için geri dönüşüm kutusu var mı? |  |  |
| Plastik şişe atıklarınızın yüzde kaçını belirlenmiş bir geri dönüşüm kutusuna attınız? | 0-100 | |
| Yaşadığınız yerde cam atıklar için geri dönüşüm kutusu var mı? |  |  |
| Cam atıklarınızın yüzde kaçını belirlenmiş bir geri dönüşüm kutusuna attınız? | 0-100 | |
